# Behavioural fever is a synergic signal amplifying the innate immune response

**DOI:** 10.1098/rspb.2013.1381

**Published:** 2013-09-07

**Authors:** Sebastian Boltaña, Sonia Rey, Nerea Roher, Reynaldo Vargas, Mario Huerta, Felicity Anne Huntingford, Frederick William Goetz, Janice Moore, Pablo Garcia-Valtanen, Amparo Estepa, S. MacKenzie

**Affiliations:** 1 Institut de Biotecnologia i de Biomedicina, Universitat Autonoma de Barcelona, Bellaterra (Barcelona) 08193, Spain; 2 Fish Biology Group, Division of Environmental and Evolutionary Biology, Institute of Biomedical and Life Sciences, Glasgow, UK; 3 Northwest Fisheries Science Centre, Seattle, WA, USA; 4 Biology Department, Colorado State University, Fort Collins, CO, USA; 5 Institute of Molecular and Cell Biology, Universidad Miguel Hernandez de Elche, Spain; 6 Institute of Aquaculture, University of Stirling, Stirling, UK

**Keywords:** behavioural fever, anti-viral response, gene–environment interaction

## Abstract

Behavioural fever, defined as an acute change in thermal preference driven by pathogen recognition, has been reported in a variety of invertebrates and ectothermic vertebrates. It has been suggested, but so far not confirmed, that such changes in thermal regime favour the immune response and thus promote survival. Here, we show that zebrafish display behavioural fever that acts to promote extensive and highly specific temperature-dependent changes in the brain transcriptome. The observed coupling of the immune response to fever acts at the gene–environment level to promote a robust, highly specific time-dependent anti-viral response that, under viral infection, increases survival. Fish that are not offered a choice of temperatures and that therefore cannot express behavioural fever show decreased survival under viral challenge. This phenomenon provides an underlying explanation for the varied functional responses observed during systemic fever. Given the effects of behavioural fever on survival and the fact that it exists across considerable phylogenetic space, such immunity–environment interactions are likely to be under strong positive selection.

## Highlights

— First observation of behavioural fever in zebrafish under a simulated viral infection,
— behavioural fever induces a major, coordinated upregulation of anti-viral genes,
— viral infection is rapidly cleared in fish expressing behavioural fever, and
— the adaptive value of behavioural fever may lie at the level of gene–environment interaction.

## 1. Introduction

The vast majority of animal species are ectothermic and can only manipulate their body temperature by behavioural means, including the choice of an appropriate environmental temperature. Whereas in endotherms a primary symptom of infection is a rapid increase in corporal temperature known as fever, ectotherms lack intrinsic thermogenesis and therefore modify body temperature by moving to warmer places, that is, they must employ behavioural fever. This phenomenon was first reported more than three decades ago in reptiles [1] and subsequently in fish [2], amphibians [3] and several invertebrates [4,5] and has been shown to increase survival [6–9]. This mirrors the well-known propensity of healthy ectotherms to use thermal gradients to attain an optimal physiological state through behavioural responses [10,11].

In endotherms, the basis of physiological regulation of body temperature has been well documented, and equivalent structures have been identified in ectothermic vertebrates, including fish (for review, see [12]). Fever is initiated by convergent signalling from the periphery of the brain by pyrogenic mediators such as prostaglandin E2 (PGE_2_) and cytokines that target the preoptical area (POA) in the anterior hypothalamus [13,14]. The immune response generates these signals through pathogen recognition receptor (PRRs) interactions, with pathogen-associated molecular patterns (PAMP) initiating both local and systemic defence responses [15]. In mammals, fever is known to promote the inflammatory response.

Less is understood about fever in ectotherms, but what is clear is that fever, whether internal or behavioural in origin, often promotes survival by enhancing defence mechanisms. However, although increased body temperature is tightly correlated with metabolic activity, the actual mechanism by which fever enhances immunity in ectothermic vertebrates remains unclear. We hypothesized that behavioural fever must be underpinned by temperature-dependent effects acting upon the transcriptome; this, in turn, specifically amplifies the immune response and therefore increases survival.

In order to test this hypothesis, we used a zebrafish model to ask the following three questions:

(1) Does a simulated viral infection cause behavioural fever? And if so:
(2) What, if any, changes occur in the brain transcriptome during the fever response?
(3) How does fever affect survival of infected hosts?

## 2. Results

### (a) Establishment of behavioural fever

**Figure 1.**
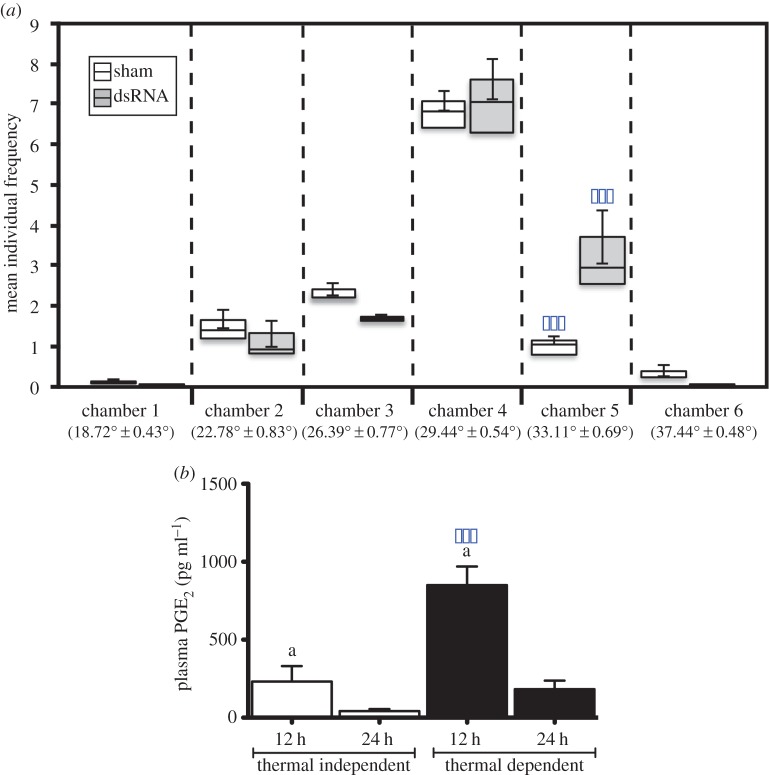
Behavioural fever in dsRNA-challenged adult zebrafish. (*a*) Frequency of chamber occupation in individual adult zebrafish (group = 10 individuals) challenged with dsRNA in a thermal gradient. Two-tailed repeated measures ANOVA; *F*_5,1190_ = 50.481, boxplots show Q1 and Q3 quartiles and mean values (*n* = 3 ± s.d., *p < 0.05; **p < 0.01; ***p < 0.001). (*b*) Plasma concentrations of PGE_2_ (pg ml^−1^) in dsRNA-challenged zebrafish (12 and 24 h) under constant conditions (T^i^) or in a thermal gradient (T^d^) (29 ± 0.5°C) *n* = 6, one-tail ANOVA; *F*_3,15_ = 17.69, (*n* = 3, mean ± s.d, *p < 0.05; **p < 0.01; ***p < 0.001). (Online version in colour.)

To test our hypothesis, we first demonstrated the existence of behavioural fever in zebrafish, *Danio rerio*. We used a simulated viral infection (synthetic double-stranded RNA (dsRNA)), in an experimental environment where fish could move freely throughout a thermal gradient. Our experimental set-up provided a gradient (35–18°C) across six contiguous linked chambers where fish (held in groups of 10) could move freely between chambers. Under constant temperature conditions (29 ± 0.5°C), individual distribution across tank chambers was not significantly different among different groups of fish. In the gradient, fish spent most time in a central chamber where mean temperature was 29.4 ± 0.54°C (see the electronic supplementary material, figure S1), the thermal preference for this species. A significant effect of the simulated viral challenge with the synthetic dsRNA was observed in the form of a mean 3 ± 0.5°C shift in thermal preference maintained over at least a 24 h period (figure 1*a*). In the vertebrates, fever is driven by peripheral pro-inflammatory cytokines produced in response to PAMPs and by subsequent prostaglandin (PGE_2_) synthesis. Measured plasma levels of PGE_2_ in synthetic dsRNA-challenged zebrafish with access to the thermal gradient were significantly higher than those of fish under conditions that limited thermal choices (figure 1*b*). Thus, increased signalling to the brain is expected to result in thermal modification by external (behavioural) or internal pyrogenesis.

### (b) The brain transcriptome and the anti-viral response

**Figure 2.**
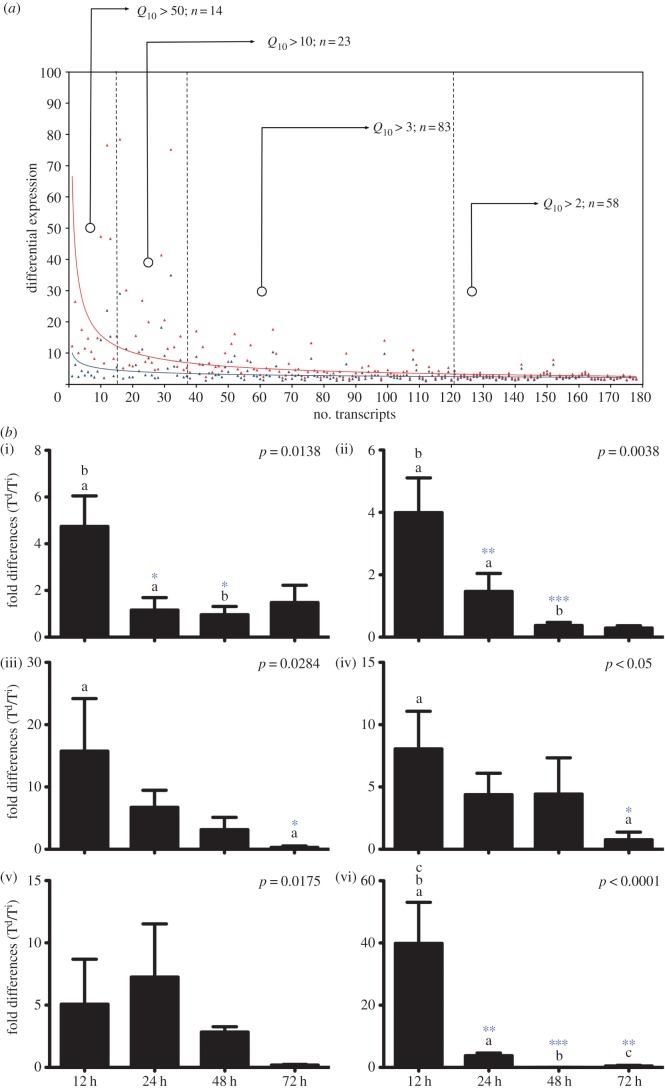
Gene–environment interaction during dsRNA-induced behavioural fever. (*a*) Differential expression levels of 156 (fold change greater than 2) dsRNA-induced transcripts common to both constant conditions (T^i^) or in a thermal gradient (T^d^). *Q*_10_ values were calculated and shown in four groupings relative to intensity. Solid curves, red (T^d^) or blue (T^i^) denote specific mRNA abundances relative to environmental condition. (*b*) qRT-PCR quantification of specific anti-viral mRNA accumulation over a 72 h time period post-dsRNA challenge. Values shown are maximal mRNA relative abundance ratio (T^d^/T^i^) in dsRNA challenged fish (*n* = 6) mean ± s.d. Two-way ANOVA: (i) Stat-1a, (ii) Stat-1b, (iii) Irf7, (iv) Gig2, (v) Trim25 and (vi) Somatostatin (interaction values). Letters represent comparisons (a,b,c) and significance is Bonferroni post-hoc test (*p < 0.05; **p < 0.01; ***p < 0.001).

Although the mechanistic basis for thermal regulation is well established, it fails to explain the underlying effect of temperature increase on the immune response. Therefore, having established the existence of behavioural fever in zebrafish, we conducted transcriptome analyses on whole brains from individual fish from four different experimental groups: synthetic dsRNA-injected fish with access to a temperature gradient (T^d^; temperature dependent), synthetic dsRNA-injected fish without access to a temperature gradient (T^i^; temperature independent), sham-injected fish in a temperature gradient (S^d^) and control, non-injected fish (C). dsRNA treatment (T^i^) and treatment + temperature (T^d^) gene expression profiles were derived by subtracting both C and S from T profiles. This allowed us to identify a core set of transcripts, produced in response to synthetic dsRNA injection, that were common to both T^i^ and T^d^ profiles (403 mRNAs *p* < 0.01; electronic supplementary material, table S1). To quantify the effect of temperature on the core response (T^d^), we calculated *Q*_10_ values for all common transcripts. Thirty-nine per cent of the core transcripts (156) exhibited *Q*_10_ values of greater than 2 in the T^d^ individuals, identifying a strong temperature-dependent effect upon transcript abundance (see figure 2*a* and electronic supplementary material, table S1). The most highly temperature-regulated groups (*Q*_10_ > 10) were almost entirely related to the anti-viral response, indicating a specific potentiation of anti-viral defence mechanisms (see the electronic supplementary material, table S2*a*). Thus, owing to increased transcription rates and de novo transcription, behavioural fever affects the anti-viral response by greatly increasing specific mRNA abundance.

To determine whether behavioural fever simply shifts the global gene expression profile by advancing or delaying the response, we compared maximal measured abundance levels of six core synthetic dsRNA-induced mRNA transcripts identified in array analyses over the initial 72 h period post-challenge. Measured transcript accumulation highlighted significant differences in maximal levels (T^d^/T^i^) and accumulation of dsRNA-induced specific mRNAs that were temperature dependent (figure 2*b*). A significant interaction was identified between temperature and time. Thus, the difference in mRNA abundance observed is not a consequence of a temporal shift; rather, it reflects a specific temperature-dependent increase in transcript abundance related to the behavioural fever response to dsRNA, suggesting increased focalization of transcription and functional specialization. This coordinated response over time shows that pyrogenesis driven by behavioural modification may strongly affect the intensity of an anti-viral immune response.

**Figure 3.**
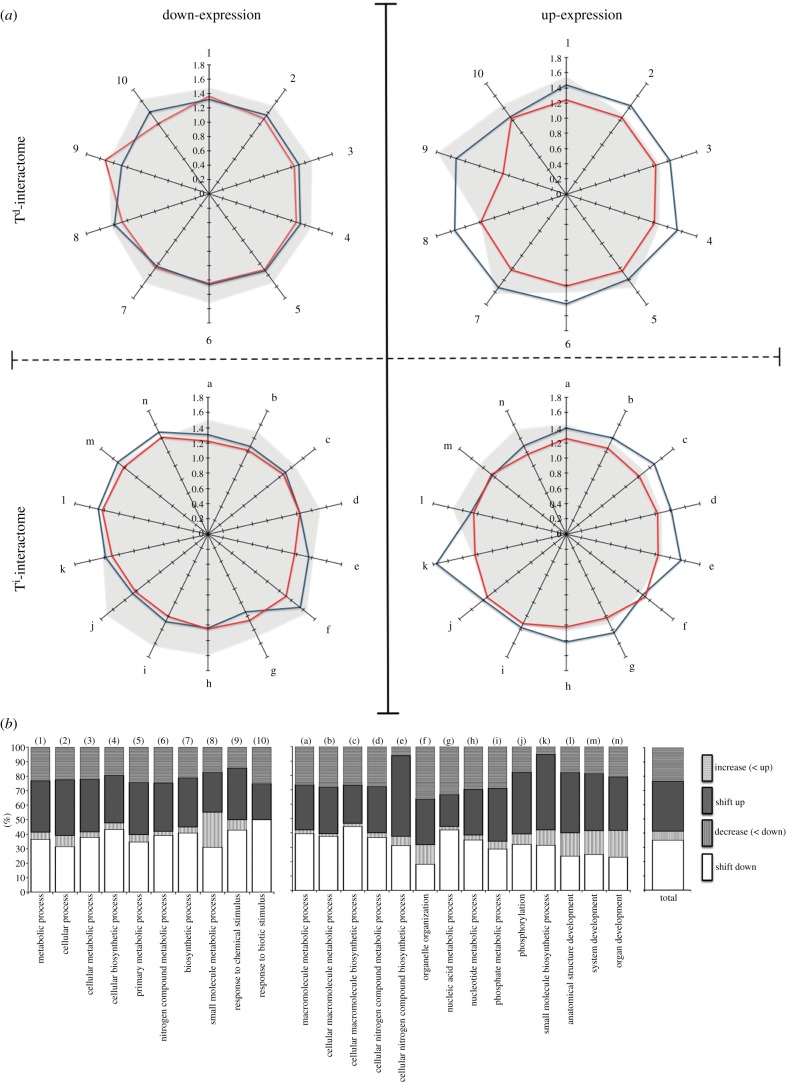
Temperature-dependent effects on transcriptome regulation. (*a*) Variation in total transcript abundance (insets; vertical axis = mean expression intensity over control interactome, one-tail ANOVA; *F*_7,99_ = 6.833, *p* < 0.0001) scores derived from T^d^ and T^i^ interactome GO clusters (*p* < 0.05). Interactome numbering (1–10) and lettering (a–n) represents T^d^ and T^i^ clusters, respectively, term description is in figure 2*b*. (*b*) Percentile representation of temperature dependent (T^d^ versus T^i^) directional shift (black and white bars represent up- and downregulated transcripts, respectively) and percentile increase of mean transcript abundance (horizontal or vertical etched bars represent % increase or decrease, respectively) in dsRNA-activated functional clusters.

The T^i^ and T^d^ experimental groups also differed significantly, both in mRNA diversity and specific transcript abundance (see the electronic supplementary material, figure S2). Fish challenged with synthetic dsRNA under T^i^ conditions increased the expressed mRNA repertoire, whereas in the T^d^ environment a smaller set of mRNAs are expressed more abundantly. In order to explore the likely impact of these mRNAs upon biological processes, we generated a core interactome [16,17] (synthetic dsRNA-specific response) and carried out Gene Ontology (GO) analysis by adding condition exclusive mRNAs (T^i^ or T^d^) to the core interactome (see the electronic supplementary material, table S2*b*). Two distinct predicted biological outcomes were obtained, describing the effects of the dynamic (T^d^) environment, in which fish could select a thermal regime, or the static (T^i^) environment, in which they could not select a thermal regime (see the electronic supplementary material, figure S3). Significantly more biological processes (*p* < 0.05) were identified under T^i^ conditions, an increase that reflected a corresponding increase in transcript diversity. In principle, thermal limitation may decrease ultimate survival by reducing the global availability of defence proteins for the immune response. To test this transcriptome–environment interaction further, we firstly calculated mean transcript abundance within GO clusters for both environmental scenarios with respect to controls (see the electronic supplementary material, table S2*b*). A strong thermal-dependent decrease upon mean synthetic dsRNA-driven transcript abundance was observed (figure 3*a*). Secondly, we measured the directional shift of regulated transcripts within GO clusters. Addition of the thermal variable identified 30 per cent of regulated transcripts within GO clusters to be directionally juxtaposed and linked to increased variation in abundance, signifying a change in functional output (figure 3*b*).

**Figure 4.**
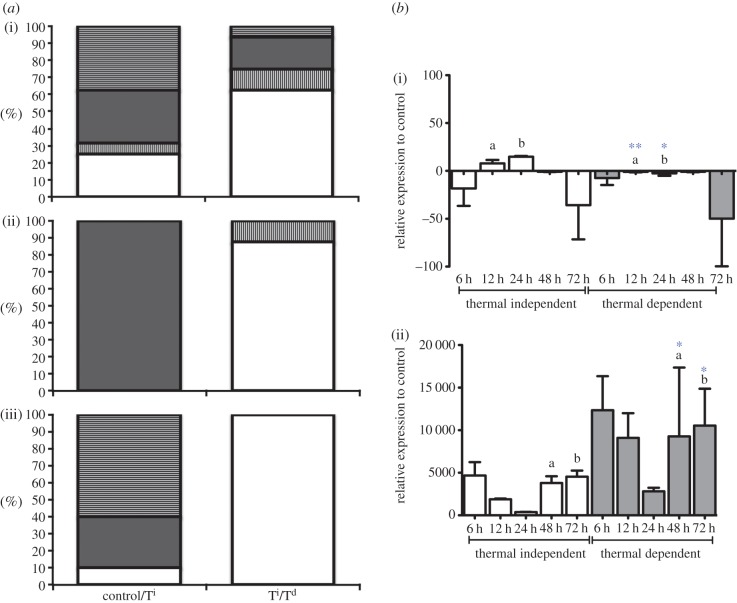
Regulation of the inflammatory reflex. (*a*) Percentile directional shift (T^d^ versus T^i^) (black and white bars represent up- and downregulated transcripts, respectively) and percentile increase of mean neuroreceptor mRNA transcript abundance (horizontal or vertical etched bars represent % increase or decrease, respectively) representative of regulatory pathways, *b*(i) α7-cholinergic receptor and *b*(ii) acetylcholinesterase mRNA abundance over a 72 h time period post-dsRNA challenge (T^d^ versus T^i^). Values shown are mRNA relative abundance ratio (*n* = 10 mean ± s.d., *T*-test, *p < 0.05; **p < 0.01; ***p < 0.001). *a*(i) nicotinic/cholinergic, *a*(ii) muscarinic and *a*(iii) adrenergic. (Online version in colour.)

To further highlight this effect, we analysed neuroregulatory components of the inflammatory reflex [18]. Previous studies suggest a central role for the cholinergic anti-inflammatory pathway in regulating systemic immune responses [19]. Under T^d^ conditions, nicotinic, adrenergic and cholinergic/muscarinic receptors are strongly inhibited, whereas the T^i^ dsRNA response is not (figure 4*a* and electronic supplementary material, table S2*c*). For example, we identified a significant inhibition of the α7 nicotinic acetylcholine receptor (α7nAChR) and a significant increase in acetylcholine esterase (AChE) transcripts, which suggests inhibition of this anti-inflammatory pathway [20] in the T^d^ environment (figure 4*b*). Thus, behavioural fever promotes the inflammatory response and shows significant similarities to the fever response in mammals at the level of regulatory system integration by promoting the inflammatory response.

### (c) Survival under viral infection

**Figure 5.**
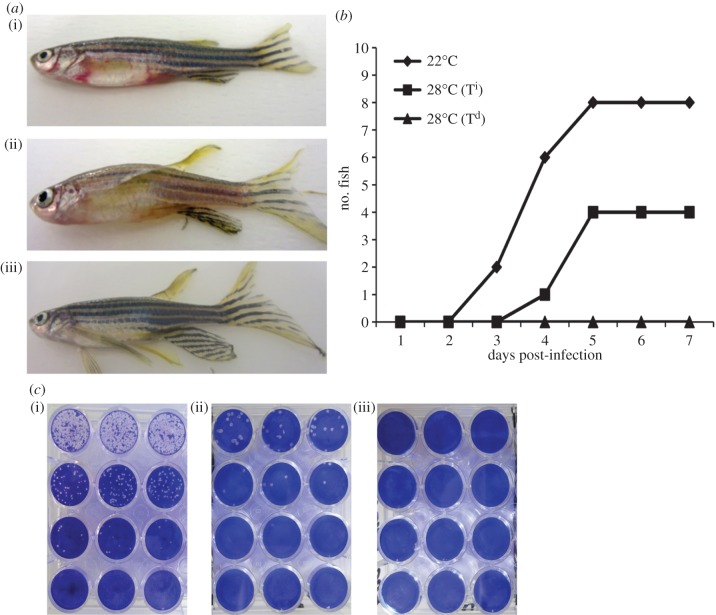
SVCV infection, clinical symptoms and virus recovery. (*a*) Representative photographs of individual zebrafish infected with SVCV 7dpi at (i) 22°C, (ii) 28°C (T^i^) and (iii) 28^o^Cd (T^d^), (*b*) appearance of clinical signs of skin haemorrhaging in SVCV-infected fish (*n* = 10) 1–7dpi in each experimental group and (*c*) plaque formation in EPC cell monolayers after infection with viral particles recovered from surviving SVCV-challenged fish 7dpi (*n* = 4) at (i) 22°C, (ii) 28°C (T^i^) and (iii) 28°C (T^d^).

Once we established a potential mechanism whereby behavioural fever potentiates the accumulation of response-specific mRNAs in the responding tissue, we sought to test the contribution of fever to survival. In the zebrafish, few relevant viral models are available; however, the Spring viraemia carp virus (SVCV) can cause lethal infections in zebrafish. In order to address our hypothesis, we therefore adapted the SVCV immersion challenge model to our fish under the thermal regimes described earlier. At 22°C, we established an LD50 at 7.1 × 10^7^ PFU ml^−1^. At 28°C, SVCV-infected fish allowed to express behavioural fever (T^d^) showed no clinical signs of infection in significant contrast to those held under constant conditions (T^i^) (see figure 5*a,b* and electronic supplementary material, table S2*d*). Importantly, we were unable to recover viral particles from the SVCV-T^d^ fish (figure 5*c*). Thus, in SVCV-infected zebrafish that are allowed to express behavioural fever all detectable SVCV virus particles are likely rapidly destroyed, as no signs of infection are appreciable.

## 3. Discussion

A change in body temperature of a few degrees centigrade above normothermia has a significant metabolic cost, since as a general rule metabolic rates increase more than 10 per cent per 1°C. Pathogen-associated molecular patterns or pathogen-induced fevers generally cause a rise of between 2°C and 5°C over normothermic conditions in both ecto- and endotherms. It is postulated that coupling of the immune response to pyrogenesis promotes survival [6–9,21]. The underlying mechanisms are unknown, although some evidence for a functional gain in defence efficacy has been reported, mainly in mammals [22–24]. However, this does not adequately explain the mechanism through which the immune response generates benefits in the face of the metabolic cost of increased body temperature. We do know that across animal phyla, innate immunity is activated by a strongly conserved set of pathogen recognition receptors [15]. This activation leads to significant transcriptome remodelling and the development of local and systemic defence responses [25,26]. Now, for the first time, our data present evidence that the adaptive value of fever may lie at the level of gene–environment interaction affecting systemic regulatory systems.

In ectotherms, the influence of environmental temperature upon the regulation of gene expression in a wide range of biological processes, including response to cold acclimation, heat stress and development has been extensively reported across different fish and invertebrate species [27–30]. It is widely accepted that acclimation to different thermal regimes strongly affects the physiological response at the level of the transcriptome. However, the majority of these studies aim towards understanding the long-term impact of temperature, e.g. climate change and more than often do not provide experimental organisms, in the case of mobile species, with a choice as experimental aims are targeting stress responses. The approach is also common in most immunological studies in fish where different yet fixed temperatures are used to assess the immune response in different species to a variety of pathogens [31,32]. Therefore, though a significant body of work addressing the effects of thermal stress is available, it is centred upon response to imposed environmental differences. The contribution of dynamic changes in thermal preference driven by behavioural choice to underpinning regulatory responses has not been investigated. Although it is known that ectotherms use thermal gradients to improve physiological performance during migration and survival through behavioural fever [10,11]. Therefore, a major difference exists between these two approaches where the latter aims to understand the optimization of regulatory responses through behavioural modification and the former aims to understand the physiological limits of the system during stress acclimation to what are often extreme environmental conditions.

We have shown that when fish are subjected to a simulated viral infection, a rise in body temperature quickly acts in synergy with the immune response to generate an extremely strong, specific anti-viral response. Our analysis highlights the importance of the temperature variable in the identification and accuracy of the measured response at both the physiological and molecular level. Integrative analysis of transcriptome data by using computational and analytical approaches helps to further support this observation by extending the interpretative value of a one-dimensional gene list into regulatory modules and interaction networks [33–35]. Comparison of T^d^ and T^i^ transcriptomes at different levels including pathway–environment interaction and interactome analysis highlights significantly different changes in the magnitude and intensity of measured responses to dsRNA. These changes can be identified at multiple levels, including single mRNAs, coordinated regulation of mRNAs, diversity of over-expressed functional groups and environment–interactome interaction providing a supporting set of inter-related analyses. Our studies suggest that behavioural fever acts to focalize the response at the level of the transcriptome and promote the emergence of a highly specific anti-viral response module, whereas the T^i^ group demonstrates a more generalized stress response based upon increased transcript diversity. The significance of this transcriptome–environment interaction is highlighted under SVCV infection where fish able to express behavioural fever rapidly combat viral infection, do not develop clinical symptoms of infection and clear replicating virus from the system. Fish held at constant temperatures provided conditions that were permissive for viral replication and as the temperature decreased viral replication and infection increased. The effect of lower temperatures on viral replication in fish has been suggested to be due to a inhibition of the immune response although at higher temperatures the immune response is considered to be more effective in viral clearance [36,37]. In our model, it is likely that the combination of both factors, optimization of thermal preference to increase the efficiency of the immune response, act in synergy to increase the efficacy of the anti-viral response. This would lead to an abrogation of viral replication hence we could not recover replicating virus from these groups of fish.

In further support of this observed synergy, we were, for the first time in a non-mammalian vertebrate, able to identify significant differences in neuroregulatory receptors and in particular components of the inflammatory reflex, α7nAChR and AChE mRNA transcripts, in the brain [38]. Increasing evidence highlights how the inflammatory response is monitored, integrated and controlled by the nervous system thus describing a tight interaction between both of these essential systems [18,19,39]. We observed a significant effect in the T^d^ environment where the majority of neuroregulatory receptor transcripts within defined functional categories were downregulated although in contrast AChE was upregulated. AChE activity has been shown to be temperature dependent in vertebrate and invertebrate ectotherms where increased T^o^C increases activity [40–42]. This suggests that behavioural fever may inhibit the anti-inflammatory reflex and increases AChE activity thus reducing inhibitory cholinergic output and providing conditions for increased anti-viral activity. This observation has strong parallels to experimental observation in mammals [19]. Furthermore, increased plasma PGE_2_ correlated to the T^d^ environment and in mammals PGE_2_ leads to fever through interaction in the preoptic area of the brain [12]. Our analysis highlights the key functions of the brain in regulating the distinct phases of fever response and is likely where the behavioural modification leading to warmth-seeking initiates. Although in mammals the beneficial or deleterious effects of fever are still debated [43,44] our results extend previous observations that behavioural fever in ectotherms has a positive adaptive value due to increased survival [8,9].

We propose that behavioural fever acts as an integrative signal that orchestrates biological output by promoting specific protein production in responding cell populations. This, in turn, leads to increased efficacy of defence traits and provides a positive adaptive value to the host. Behavioural fever is widespread among animals and may reflect an ancient mechanism conserved across evolutionary time that affects host and pathogen fitness. The observed gene–environment synergy leading to increased survival has implications towards understanding the molecular basis of disease resistance in ectotherms.

## 4. Experimental procedures

### (a) Animals

Zebrafish (*Danio rerio*) (0.93 ± 0.22 g and 43.73 ± 2.44 mm; *n* = 206) were purchased from a commercial supplier (Piscicultura Superior SL, Barcelona, Spain) and held in a recirculating aquarium rack system (zfbiolabs) at the Universitat Autònoma de Barcelona, Spain. Fish were kept at 28°C on a 14 L : 10 D photoperiod cycle, and fed twice a day (0.5% body weight per day) on a wet commercial diet (zfbiolabs). Water quality indicators (dissolved oxygen, ammonia, nitrite, pH) were analysed weekly to maintain recommended quality.

### (b) Behaviour studies

The experimental thermal gradient tank is a 2.36 m^3^ tank (105 × 15 × 15 cm) divided with five transparent Plexiglas screens to create six equal interconnected chambers. Each screen has a hole at the centre (3 cm diameter; 10 cm from the bottom) to allow connection between chambers. Three video cameras provide continuous monitoring of each tank chamber. During an experiment, temperatures were recorded for 10 s every 15 min throughout daylight hours (12 h = 48 recorded events). Four groups of fish (*n* = 10 for each group) were introduced into chamber 4 in the evening and filming began at 6.00 the next day, providing a 12 h acclimation period. Thermal gradients were achieved with a mean difference in temperature of 18.72°C between chambers 1 and 6 by simultaneously heating chamber 6 (mean temperature = 37.44 ± 0.48°C) and cooling chamber 1 (mean temperature = 18.76 ± 0.36°C). All temperatures were recorded each day at the same time of the day. Experimental groups were (i) control with no gradient, (ii) intraperitoneal sham injection with saline (5 µl of 1 × PBS) in a temperature gradient, (iii) intraperitoneal injection with dsRNA (poly (I : C); polyinosine : polycytidylic acid) (10 μg kg^−1^) in a temperature gradient, and (iv) intraperitoneal injection with dsRNA (poly (I : C); 10 μg kg^−1^) under constant normothermic (preferred temperature) conditions. Two-way repeated measures ANOVA, SPSS statistics 17 package, was used to compare chamber occupation frequencies between different experimental conditions.

### (c) Measurement of plasma prostaglandin

Blood plasma was obtained from individual zebrafish and stored at −80°C until use. Measurement of plasma PGE_2_ levels was carried out using a commercial monoclonal EIA according to the manufacturer's instructions (Cayman). The prostaglandin kit detection limit was 8 pg ml^−1^. Data obtained were analysed using the SPSS statistics 17 package using one-way ANOVA.

### (d) Spring viraemia carp virus studies

The fish cell lines EPC (Epithelioma papulosum cyprinid; fathead minnow (*Pimephales promelas*) purchased from the American Type Culture Collection (ATCC no. CRL-2872) and ZF4 (zebrafish embryonic fibroblast) purchased from the ATCC (CRL-2050) were used. EPC and ZF4 cell lines were maintained at 28°C in a 5 per cent CO_2_ atmosphere with RPMI-1640 Dutch modified (Gibco, Invitrogen corporation, UK) cell culture medium containing 10 per cent fetal calf serum (FCS) (Sigma, St Louis, USA), 1 mM pyruvate (Gibco, Invitrogen Corporation, UK), 2 mM glutamine (Gibco), 50 µg ml^−1^ gentamicin (Gibco) and 2 µg ml^−1^ Fungizone. The isolate 56/70 of SVCV isolated from carp was propagated in ZF4 cells at 22°C (adapted from [27]). Supernatants from SVCV-infected cell monolayers were clarified by centrifugation at 4000*g* during 30 min and kept in aliquots at –70°C. Clarified supernatants were used in the experiments [45].

### (e) Spring viraemia carp virus challenge

*In vivo* infection of zebrafish was done by immersion using dechlorinated water from stock tanks following protocols previously described for the viral haemorrhagic septicaemia fish rhabdovirus [46]. For immersion challenge assays, clarified supernatant from SVCV-infected zf4 cell monolayers (7.1 ± 2 × 10^7^ PFU ml^−1^) was added to water in 4 l water tanks containing the fish (*n* = 30). After 90 min, fish were separated into three groups (*n* = 10 each): 22°C, 28°C^i^ (constant temperature) and 28°C^d^ (temperature gradient). The fish were scored daily for a 7-day period; we recorded abdominal distension, exophthalmia, impaired swimming and skin/fin base haemorrhages. In the thermal gradient, behavioural data were recorded as described earlier throughout the challenge experiments. Three independent experiments were carried out with a total of 90 fish.

### (f) Spring viraemia carp virus recovery and titration

Four surviving fish from each group were randomly sampled at 7 days post-infection (dpi), the end of each experiment. Prior to tissue removal, all fish were exposed to an overdose of tricaine (MS222, Sigma-Aldrich). Fin bases, spleen, gills and head kidney were excised from the fish and disrupted using a sterile nylon cell strainer (BD Falcon MA, USA) and a pestle and subsequently passed through 0.2 μm sterile filters to remove bacterial contamination. Pooled cell samples from each group of four fish were resuspended in 3 ml of RPMI (Roswell Park Memorial Institute) cell culture medium prepared as described earlier. Virus titres recovered from fish in each experiment were determined by plaque assays. Different dilutions of the virus were used to infect EPC cell monolayers in 24-well plates. Infections were carried out at 22°C in RPMI cell culture media with 2 per cent FCS for 1.5 h. Then, the media were removed from each well and covered with a solution of RPMI cell culture medium with 2 per cent FCS and a 2 per cent aqueous solution of methyl cellulose (SIGMA, St Louis, USA). Cell plates were kept at 22°C for 5 days and the media with methyl cellulose removed. All wells were stained with crystal violet–formalin to count plaques.

### (g) RNA isolation

Total RNA was extracted from individual zebrafish brains using 0.3 ml of TriReagent (Molecular Research Center) following the manufacturer's instructions. RNA concentration was quantified (Nanodrop ND-1000) and RNA integrity and quality assessed (Bioanalyzer 2100, Agilent Technologies). The RNA integrity number (RIN) was calculated for each sample and only RNAs with an RIN number more than 7 were processed. RNA (1 μg) was used to synthesize cDNA with SuperScript III Transcriptase (Invitrogen) and oligo-dT primer (Promega).

### (h) RNA labelling and microarray hybridization

RNA samples were grouped into pools of three individual brains for each treatment. Microarray hybridizations were performed using the Zebrafish v. 2 (ID 026437) 4 × 44 K Agilent oligonucleotide microarray. A loop microarray design approach using single colour labelling was used for the study. Standard methods were used for all processes according to manufacturer's instructions (Agilent Technologies). Briefly, each amplified and labelled sample was hybridized at 65°C for 17 h. Microarrays were scanned and one-channel TIFF images (Feature Extraction software v. 10.4.0.0) were imported into GeneSpring software (GX v. 11.0). Microarray data are described in accordance with MIAME guidelines and have been submitted to Gene Expression Omnibus (GEO) database with the no. GSE32636.

### (i) Microarray analysis

Standard analytical methods were applied to analyse data obtained. Briefly, array normalization (percentile shift normalization) was carried out and data filtered by standard deviation expression among groups (filter by expression). Signal intensities for unique probes within a probe set were averaged to obtain an expression value (median) for the probe set (Gene-level analysis). We defined an ‘expressed’ transcript as that with a log2-transformed expression value greater than or equal to 1 in one or more brain samples. Statistical tests were implemented in the GeneSpring software GX v. 11.0 used to select transcripts differentially expressed (*p* < 0.001) between control and treatments. One-way ANOVAs were used to identify significant differences between treatments. Principal component analysis was used to describe differences among groups and the correlation matrix and Kaiser–Meyer–Olkin contrast determined for T^i^ and T^d^ conditions (see the electronic supplementary material, figure S2).

### (j) Interactome analyses

Visualization of interactions and overlays of expression profiles was carried out using Cytoscape v. 2.8.2. (http://www.systemsbiology.org). The interactome network was obtained from all interactions with a FBS > 6. The interactome backbone contains 5760 nodes (protein–protein and protein–DNA interactions) and 99 573 relationships between these proteins (interactions) (see the electronic supplementary material, table S4). The designation of protein properties was drawn from Alexeyenko *et al*. [17]. And NCBI gene name attributes were used to unify the protein list and were imported through the Biomart plugin [47]. The network for the ZFcorePIC primary interactors direct neighbours (level 2) was built from within *Danio rerio*_fever interactome. The group was selected and served to populate a sub network with the first neighbours and adjacent edges into a unified interactome. Topological analysis of individual and combined networks was performed with Network Analyzer [48], and jActiveModules v. 2.2 [34] was used to analyse network characteristics. GO analysis was conducted with the Biological Network Gene Ontology (BinGO, v. 2.0) plugin [49] and GOlorize tools [50] used for statistical evaluation of groups of proteins with respect to the present annotations available in the Gene Ontology Consortium (http://www.geneontology.org). GO overrepresentation was calculated using the hypergeometric test with Benjamini and Hochberg false discovery rate (FDR) multiple testing correction and significance (*p*_FDR_ < 0.05).

### (k) Real-time quantitative PCR

Standard SYBR-green-based methodology was used for rtqPCR studies. Briefly, 2 μg of total RNA was used for cDNA synthesis (SuperScript III, Invitrogen) and subsequently diluted with nuclease-free water to 1 ng μl^−1^ cDNA. Gene-specific high-melting temperature primers for genes of interest were designed using NCBI/Primer-BLAST suite (http://www.ncbi.nlm.nih.gov/tools/primer-blast/) (see the electronic supplementary material, table S2*e*). PCR reactions were conducted on an ABI 7900 Sequence Detection System (Applied Biosystems) using a hot start SYBR-green-based method (Fast SYBR Green Master Mix, ABI) followed by melting curve analysis to verify specificity of the product. All transcripts were normalized to the housekeeping gene *18s.* Quantitative expression data were examined either by two-way ANOVA using the SPSS 17 statistical software and the differences were reported as either exact *p* values or alpha values (*p* < ) or as two-tailed *t*-tests with alpha values.

## Notes

The experimental protocols used for fish sampling have been reviewed and approved by the Ethics and Animal Welfare Committee of the Universitat Autònoma de Barcelona, Spain.
